# Transcriptional Dysregulation and Post-translational Modifications in Polyglutamine Diseases: From Pathogenesis to Potential Therapeutic Strategies

**DOI:** 10.3389/fnmol.2018.00153

**Published:** 2018-05-15

**Authors:** Chunchen Xiang, Shun Zhang, Xiaoyu Dong, Shuang Ma, Shuyan Cong

**Affiliations:** Department of Neurology, Shengjing Hospital of China Medical University, Shenyang, China

**Keywords:** polyglutamine diseases, Huntington’s disease, transcriptional dysregulation, post-translational modification, histone deacetylase inhibitor

## Abstract

Polyglutamine (polyQ) diseases are hereditary neurodegenerative disorders caused by an abnormal expansion of a trinucleotide CAG repeat in the coding region of their respective associated genes. PolyQ diseases mainly display progressive degeneration of the brain and spinal cord. Nine polyQ diseases are known, including Huntington’s disease (HD), spinal and bulbar muscular atrophy (SBMA), dentatorubral-pallidoluysian atrophy (DRPLA), and six forms of spinocerebellar ataxia (SCA). HD is the best characterized polyQ disease. Many studies have reported that transcriptional dysregulation and post-translational disruptions, which may interact with each other, are central features of polyQ diseases. Post-translational modifications, such as the acetylation of histones, are closely associated with the regulation of the transcriptional activity. A number of groups have studied the interactions between the polyQ proteins and transcription factors. Pharmacological drugs or genetic manipulations aimed at correcting the dysregulation have been confirmed to be effective in the treatment of polyQ diseases in many animal and cellular models. For example, histone deaceylase inhibitors have been demonstrated to have beneficial effects in cases of HD, SBMA, DRPLA, and SCA3. In this review, we describe the transcriptional and post-translational dysregulation in polyQ diseases with special focus on HD, and we summarize and comment on potential treatment approaches targeting disruption of transcription and post-translation processes in these diseases.

## Introduction

Nine genetic neurodegenerative diseases are caused by the expansion of CAG repeats in seemingly unrelated genes. Spinal and bulbar muscular atrophy (SBMA) reveals an X-linked pattern of inheritance while others are inherited in an autosomal dominant fashion. Neurodegenerative diseases are a group of devastating disorders presenting as movement and/or cognitive impairment affecting a certain population of neurons within the central nervous system. PolyQ diseases mainly display progressive degeneration of the brain and spine, and in the case of SCA7, the macula and retina. These diseases have a striking genotype–phenotype correlation. The length of the CAG repeat is strongly positively correlated with disease severity and time of disease onset. As these diseases progress, the extensive neurodegeneration results in cell apoptosis.

Some groups have emphasized the regulatory roles of polyQ protein and different transcription factors, coactivators, or transcriptional regulators. Post-translational protein modifications can affect transcription and also play a major role in the disease pathogenesis. However, the mechanisms underlying transcriptional and post-transcriptional dysregulation in polyQ diseases remain incomplete. Several studies have concentrated on pharmacological interventions in several animal models of polyQ diseases. However, further studies are needed before these treatments can be used clinically.

In this review, we focus on the transcriptional and post-translational dysregulation in polyQ diseases. We present the existing evidence for the regulation of transcription factors and co-activators in polyQ diseases and further discuss the role of post-translational regulation in polyQ pathogenesis. We mainly discuss the regulation of Huntington’s disease (HD), which is the best-known polyQ disease. Finally, we summarize the latest therapeutic strategies for polyQ diseases, based on selective targeting of the transcriptional and post-translational dysfunctions. Challenges and further directions regarding these novel approaches will be discussed in the last part of the review.

## Huntington’s Disease

Huntington’s disease is a fatal hereditary neurodegenerative disorder characterized by progressive motor and cognitive deficits. The pathogenesis is associated with an abnormal expansion in the number of glutamine residues located in the amino (N) terminus of huntingtin (Htt), a very large protein found mainly in the cytoplasm (The Huntington’s Disease Collaborative Research Group, 1993). PolyQ expansion in mutant huntingtin (mHtt) leads to its aberrant proteolytic cleavage, generating N-terminal fragments that are translocated to the nucleus. Nuclear aggregation of mHtt is involved in neuronal loss in the striatum and cortex (DiFiglia et al., 1997).

### Transcription

Many studies have shown that the nuclear effects of mHtt might be associated with its interaction with a number of transcriptional regulators, including the cAMP response element-binding protein (CREB), CREB binding protein (CBP), repressor element 1 (RE1)-silencing transcription factor (REST), and other factors (Kumar et al., 2014; Valor, 2015).

Until now, scientists have failed to find any effective treatments for HD (Wild and Tabrizi, 2017). However, some preclinical studies have indicated approaches with some potential therapeutic value in the prevention and treatment of HD (**Table 1**).

**Table 1:** Potential targets for prevention and treatment in several HD models.

Targets	Interventions	Techniques	Key findings	Reference
PDE inhibitor	Rolipram	Striatal interneurons and spinal neurons in R6/2 HD mice model	Rolipram, a PDE type IV inhibitor, increased the levels of activated CREB and BDNF	DeMarch et al., 2008; Giampa et al., 2009
	Sildenafil, vardenafil	3-NP mice model	Sildenafil and vardenafil, PDE 5 inhibitors, modulate CREB and BDNF and protect rats	Puerta et al., 2010
	TP-10	Medium spinal neurons, striatal and cortical cells in R6/2 HD mice model	TP10 was a PDE10A inhibitor and reduced striatal pathology	Giampa et al., 2010; Leuti et al., 2013
	Papaverine	Hippocampus in R6/1 HD mice model	Papaverine inhibited PDE10 and improve cognition function	Giralt et al., 2013
	TAK-63	Medium spiny neurons in R6/2 HD mice model	TAK-63 inhibited PDE10A, reduced striatal neurodegeneration and ameliorated behavioral deficits	Harada et al., 2016
	PF-920	Q175 mice	Early chronic administration showed improvementsafter symptom onset	Beaumont et al., 2016
REST	REST decoy oligonucleotides	HD cellular model	Transfection of REST decoy oligonucleotides restore BDNF expression	Soldati et al., 2011
	Dominant negative form of REST (DN:REST)	Motor cortex in BACHD and N171-82Q HD mice model	Overexpressing of DN:REST restores the mRNA and protein levels of BDNF	Conforti et al., 2013a
	Quinone-like compound 91 (C91)	Htt-knockdown zebrafish	C91 induces REST target genes expression and increases BDNF mRNA in the presence of mHtt	Conforti et al., 2013b
PCG agonist	Rosiglitazone	Striatal cells in N171-82Q HD mice	Rosiglitazone attenuated mHtt-induced toxicity and improved motor function	Jin et al., 2013
	Thiazolidinedione	Adipocytes of R6/2 HD mice	Thiazolidinedione rescued motor deterioration and formation of mHtt aggregates	Chiang et al., 2010
	Bezafibrate	Brown adipose tissue in R6/2 HD mice/striatum in the BACHD mice	Bezafibrate rescued neuropathologic features and increased life expectancy	Johri et al., 2012; Chandra et al., 2016
	Adipose-derived stem cells (Asc)	R6/2 mice-derived neuronal cells and R6/2 mice model	Exosome from Asc upregulated PCG-1 alpha and phosphorylated CREB levels, reducing mHtt aggregation	Im et al., 2013; Lee et al., 2016
Sp1	Mithramycin	*Drosophila* and mouse model of HD	A gene-selective sp1 inhibitor could increase the lifespan	Ferrante et al., 2004; Sleiman et al., 2011
NF-κB inhibitor	EVP4593	Striatum from YAC128 mice	EVP4593, an NF-κB pathway inhibitor, protected medium spinal neurons	Wu et al., 2011
	Natrium diethyl dithiocarbamate trihydrate (NDDCT)	3-NP-induced mice model	NF-κB inhibitor, NDDCT, attenuated toxicity	Gupta and Sharma, 2014
	Ethyl pyruvate (EP)	Striatum in 3-NP -induced mice model	EP inhibited NF-κB pathway and increased survival rate	Jang et al., 2014
	Sulforaphane	3-NP-induced mice model	Sulforaphane inhibited NF-κB pathway and attenuated toxicity	Jang and Cho, 2016
HDAC inhibitor	SAHA	*Drosophila* HD model	SAHA, HDAC inhibitor, slowed the pathogenesis of HD	Steffan et al., 2001
	LBH589	R6/2 and full-length CAG140 knock-in HD mice models	Non-selective HDAC inhibitor, LBH589, improved motor performance	Chopra et al., 2016
	Phenylbutyrate	N171-82Q HD mice model	HDAC inhibitor, phenylbutyrate, ameliorated degeneration	Gardian et al., 2005
	Sodium butyrate (SBP)	R6/2 mice	HDAC inhibitor, SBP, modulated transcription and extended survival	Ferrante et al., 2003
	SBP	Phase II clinical trial in HD subjects	SBP treated with 12–15 g/day was safe and well-tolerated	Hogarth et al., 2007
	RGFP966	N171-82Q HD mice model	Selective HDAC3 inhibitor, RGFP966, activated glial cell and astrocyte	Jia et al., 2016
	4b	N171-82Q HD mice model	4b, selectively targeting HDAC1 and HDAC3, prevented formation of mHtt	Jia et al., 2012
Sirt1 activator	Resveratrol (RESV)	YAC128 mice model and N171-82Q HD mice model	RESV, the activator of Sirt1, decreased H3 acetylation and improved motor coordination	Ho et al., 2010; Naia et al., 2016
	SRT2104	N171-82Q HD mice model	SRT2104, sirt1 activator, improved motor function and extended life span	Jiang et al., 2014
Sirt1 inhibitor	NAM	B6.HD6/1 mice model	NAM, sirt1 inhibitor, could restore BDNF expression	Hathorn et al., 2011
	Selisistat	*Drosophila* and R6/2 HD mice model and HEK293 cell	Selisistat rescued neuronal degeneration and extended lifespan	Smith et al., 2014
	Selisistat	Early stage HD patients	Selisistat were safety, well-tolerated, and no beneficial effects on clinical outcome	Süssmuth et al., 2015
Sirt2 inhibitor	AK-7	R6/2 HD mice model	AK-7, the sirt2 inhibitor, extended survival	Chopra et al., 2012
	MIND4	In *ex vivo* brain slice and *in vivo Drosophila* HD model	Bioactive sirt2 inhibitor, MIND4’s neuroprotective	Quinti et al., 2016
Methylation	Protein arginine methyltransferase 5 (PAMT5)	Primary cortical neurons in HD cellular model	Compensation of PRMT5 deficiency reversed the toxic effects of mHtt	Ratovitski et al., 2015

#### CREB and CBP

**FIGURE 1:**
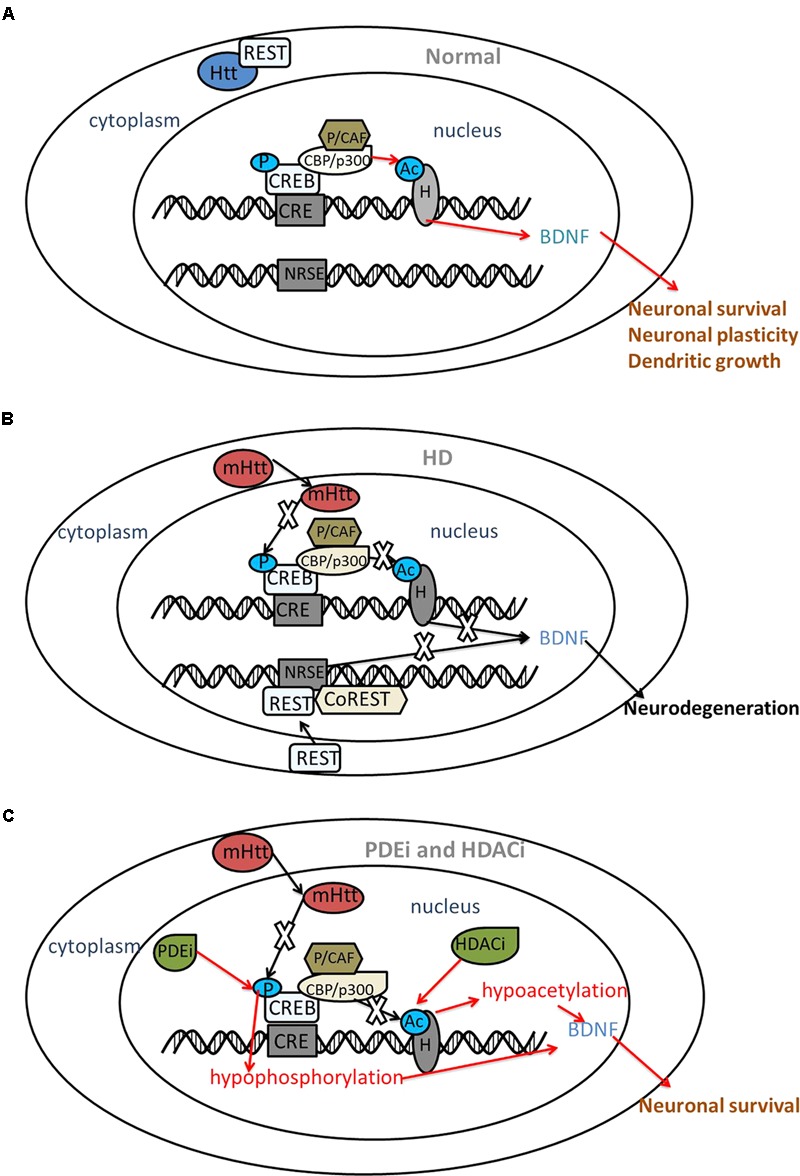
The interaction between CREB, REST and mutant huntingtin. *Red arrows indicate the protective roles and black arrows indicate the disturbed roles.* **(A)** Under normal circumstances, Htt interacts with REST, a transcriptional repressor within the cytoplasm. Htt does not interfere with phosphorylation (P) of CREB or acetyltransferase (Ac) activity of CBP; thus, acetylation of histones (H) results in transcriptional activation followed by the expression of neuroprotective genes, such as BDNF. **(B)** In HD, mHtt inhibits the phosphorylation of CREB and the acetyltransferase activity of CBP. REST translocates to the nucleus and binds with NRSE, repressing BDNF transcription. As a result, neuronal death might occur. **(C)** PDE inhibitors (PDEi) and HDAC inhibitors (HDACi) increase the phosphorylation of CREB and acetyltransferase activity of histones, restoring the expression of BDNF and displaying a neuroprotective effect in HD. BDNF, brain-derived neurotrophic factor; CRE, cAMP response element; CREB, CRE-binding protein; CBP, CREB binding protein; P/CAF, p300/CBP-associated factor; HD, Huntington’s disease; HDAC, histone deacetylases; mHtt, mutant huntingtin; NRSE, neuron restrictive silencer element; PDE, phosphodiesterase; REST, repressor element 1 (RE1)-silencing transcription factor; CoREST, co-repressor of REST.

cAMP response element-binding protein is a member of the basic leucine zipper family of transcription factors and regulates several neuroprotective processes. CREB is phosphorylated at serine 133 (Ser133), and then recruits with transcriptional co-activators CBP and p300 to activate transcription (Chrivia et al., 1993). Several groups have reported that CREB plays an important role in the pathology of HD. Choi and his group found that loss of CREB function precedes cell death in a chemical and transgenic mice model of HD. They reported that phosphorylation of CREB in the striatum is potently repressed in the 3-nitropropionic acid (3-NP) mouse model, which is often used to model HD pathology (Choi et al., 2009). A flow cytometry study of neuroblastoma cells with mHtt showed that the toxicity of mHtt impairs baseline CREB signaling, and triggering CREB signaling rescues this effect (Moily et al., 2017). Reduced CREB phosphorylation may be related to repressed brain-derived neurotrophic factor (BDNF) in the HD mouse cortical cell model (Tao et al., 1998). BDNF plays a neuroprotective role in both cellular and mouse models of HD, and its overexpression slows the progression of HD pathogenesis (Zuccato and Cattaneo, 2009; Plotkin et al., 2014). BDNF-overexpressing neural progenitors promote recovery in the R6/2 and N171-82Q mice models of HD (Zimmermann et al., 2016). The suppression of CREB-dependent transcription and the cell death induced by polyQ stretches are restored by co-expressing TAFII130 [TBP-associated factor (TAF)] (Shimohata et al., 2000) Furthermore, mHtt knockdown with shRNA prevents transcriptional repression of CREB in a HD cell model (Chaturvedi et al., 2012). Wild-type Htt (wHtt) overexpression increases activation of CREB selectively in striatal neurons (Buren et al., 2014) (**Figure 1**).

However, Obrietan and Hoyt (2004) revealed an increase in phosphorylation of CREB in several brain areas (striatum, hippocampus, and cortex) in a HD mice model.

CREB binding protein, a histone acetyltransferase (HAT), is depleted from its normal nuclear location and is present in polyQ aggregates in HD cell models, HD transgenic mice, and the human HD postmortem brain (Nucifora et al., 2001). Overexpression of CBP rescues polyQ-induced neuronal toxicity in a *Drosophila* model (Taylor et al., 2003), whereas partial depletion of CBP decreases life expectancy in the HD mouse model (Klevytska et al., 2010). However, other studies have reported no difference in CBP expression or localization in a HD mice model (Obrietan and Hoyt, 2004). Jiang et al. (2006) explained that the disease phenotypes seen in the transgenic mouse models might not fully mimic those observed in patients with HD.

mHtt inhibits the acetyltransferase activity of p300, p300/CBP-associated factor (P/CAF), as well as CBP (Steffan et al., 2001). However, other results show that mHtt specifically represses the transcriptional activity of CBP but not p300 both at the early and late time points, via multiple mechanisms (Cong et al., 2005).

Transducers of regulated CREB activity (TORCs) expression decreases in a HD cellular model, in the striatum of a transgenic mice model, and in postmortem HD striatal tissue. TORCs overexpression increases CREB mRNA and protein levels, as well as the resistance of striatal cells to 3-NP toxicity. mHtt interferes with the TORC1-CREB interaction to repress BDNF transcription (Chaturvedi et al., 2012).

Inhibiting phosphodiesterases (PDEs), which are enzymes responsible for cAMP/cGMP degradation, is responsible for increasing CREB phosphorylation and thus playing a neuroprotective role in HD. Rolipram, a PDE type 4 (PDE4) inhibitor, increases the levels of activated CREB and BDNF in the striatal spiny neurons of the R6/2 HD mouse model (DeMarch et al., 2008). The potential therapeutic value of PDE4 inhibitors might be associated with preventing CBP sequestration (Giampa et al., 2009). The PDE5 inhibitors sildenafil and vardenafil protect rats against 3-NP neurotoxicity by modulating CREB and BDNF (Puerta et al., 2010). PDE10A inhibitors, including TP10, papaverine, TAK-063, and PF-02545920 play neuroprotective roles in the pathogenesis of HD as well (Giampa et al., 2010; Giralt et al., 2013; Leuti et al., 2013; Beaumont et al., 2016; Harada et al., 2016).

In conclusion, phosphorylation of CREB was repressed, while overexpression of CBP rescued polyQ-induced toxicity. Inhibiting PDE has been confirmed by several groups to play a therapeutic role in HD.

#### Repressor Element 1-Silencing Transcription Factor (REST)

(RE1)-silencing transcription factor, also known as neuron-restrictive silencing factor (NRSF), is a repressor of neuronal gene transcription in non-neuronal lineages that binds to a 21-bp DNA element, RE-1/neuron restrictive silencer element (NRSE), thereby activating its silencing activity. In the presence of wHtt, REST is tethered in the cytoplasm, but REST translocates to the nucleus in the presence of mHtt where it represses transcription of its target genes, including the BDNF gene (Zuccato et al., 2003; Zuccato and Cattaneo, 2007). REST/NRSF occupancy increases at the RE1/NRSE site in lymphocytes from patients with HD (Marullo et al., 2010). Low levels of cytoplasmic REST are detected in neurons from patients with HD. However, no direct relationship between decreased neuronal REST expression and disease grade has been observed (Schiffer et al., 2014) (**Figure 1**).

miR-124, which is upregulated by REST, is downregulated and then increases BDNF protein levels, playing a neuroprotective role in the striatum and the cortex of the R6/2 HD mouse model, a “truncated” Htt mouse model of HD (Johnson et al., 2008; Liu et al., 2015).

Several groups have confirmed that the REST/NRSF-RE1/NRSE system might be acting as a pharmaceutical target. Transfection of REST decoy oligonucleotides in a cellular model of HD restores expression of REST target genes, such as BDNF (Soldati et al., 2011). Overexpressing the dominant-negative form of REST (DN: REST) in the motor cortex of two HD transgenic mouse models (BACHD and N171-82Q) restores BDNF mRNA and protein levels; therefore, reversing the repressive effects of REST (Conforti et al., 2013a). Quinone-like compound 91 (C91) induces the expression of REST target genes in Htt-knockdown zebrafish and increases BDNF mRNA in the presence of mHtt (Conforti et al., 2013b).

In brief, REST and its target gene transcription play important roles in the progression of HD. Molecules able to affect REST/NRSF nuclear translocation, DNA binding or, more generally, formation of the REST/NRSF transcriptional complex, may be regarded as potential drug design targets for HD.

#### Peroxisome Proliferator-Activated Receptor Gamma Coactivator-1α

Peroxisome proliferator-activated receptor gamma coactivator-1α (PGC-1α) is a transcriptional coactivator that regulates several metabolic processes, including mitochondrial biogenesis and oxidative biogenesis. Reduced full-length PGC-1α expression has been observed in transgenic mouse models of HD and in postmortem brain tissue from HD patients (Cui et al., 2006; Weydt et al., 2006), in muscle biopsies from HD patients, and in human HD myoblast cultures (Chaturvedi et al., 2009). mHtt represses CRE-mediated transcription of PGC-1α by interfering with the CREB/TAF4 transcriptional pathway in striatal neurons (Cui et al., 2006). However, levels of the N-truncated splice variant of PGC1α (NT-PGC1α) are significantly upregulated in the human HD brain, human HD myoblasts, mouse HD models, and mouse HD striatal cells (Johri et al., 2011). PGC-1α knockout mice exhibit developmental myelination deficits, contributing to the white matter atrophy in HD (Xiang et al., 2011).

Overexpression of PPARGC1A, the gene encoding for PGC-1α, rescues HD neurological phenotypes and neurodegeneration (La Spada, 2012). The PPARGC1A single nucleotide polymorphism (SNP) (rs7665116) was associated with a delay in the onset of HD in an Italian cohort (Weydt et al., 2009), while other results failed to find any association in a larger European cohort (Soyal et al., 2012). The rs2970870 SNP is related to earlier onset HD (Che et al., 2011). One study demonstrated that regulation of the coding variant (rs3736265) is associated with a male-specific earlier onset HD (Weydt et al., 2014).

The PGC-1α signaling pathway is now increasingly being recognized as an important therapeutic target for HD. Treatment with rosiglitazone, an agonist of peroxisome proliferator-activated receptor gamma (PPARγ), could prevent the reduction in PGC-1α and rescue BDNF deficiency. These effects might be related to a beneficial role in rescuing motor function in the HD mouse brain (Jin et al., 2013). The PPARγ agonist thiazolidinedione produces a beneficial effect in the R6/2 HD mouse model (Chiang et al., 2010). Bezafibrate, a pan-PPAR agonist, increases expression of PGC-1α and downstream target genes, reduces oxidative damage, improves behavioral deficits and striatal atrophy, and increases life expectancy in the R6/2 HD mouse model (Johri et al., 2012). Another study also found a similar result in a “full-length” Htt mouse model called BACHD mice (Chandra et al., 2016).

One study revealed that exosomes from adipose-derived stem cells decrease mHtt aggregates in R6/2 HD mice-derived neuronal cells, which might be related to upregulation of PGC-1α and phosphorylated CREB (Lee et al., 2016). Similar results were reported by another group (Im et al., 2013). Cytotoxicity and apoptosis are ameliorated by overexpressing miR-196a through upregulation of CBP and PGC-1α in HD monkey neural progenitor cells (Kunkanjanawan et al., 2016).

In conclusion, PGC-1α expression decreases in several HD models, while several PPARGC1A SNPs are related to earlier or delayed onset of HD. Several potential treatments associated with preventing the reduction in PGC-1α may be helpful in the intervention of HD.

#### Specificity Protein 1

Specificity protein 1 (sp1) contains three zinc-finger motifs and its C-terminal domain interacts with other transcription factors in a synergistic manner to control gene expression in a temporal and spatial manner (Zhai et al., 2005).

Several groups have reported the neuroprotective role of sp1. Dunah and his group demonstrated that co-expression of sp1 and TAFII130 decreases transcriptional dysfunction of the dopamine D2 gene caused by mHtt, as well as protects cultured wild-type and HD transgenic mice striatal cells from mHtt-induced cellular toxicity (Dunah et al., 2002). Chen-Plotkin et al. (2006) also reported that binding of sp1 to the dopamine D2 gene decreases in HD mice, striatal HD cells, and in the postmortem HD brain. mHtt was also found to inhibit sp1-dependent transcription in postmortem brain tissues of both presymptomatic and patients affected with HD, as well as in cultured HD cell models (Zhai et al., 2005). Furthermore, overexpression of sp1 reduces the cellular toxicity and neurite extension defects caused by intracellular mHtt (Li et al., 2002). Wang et al. (2012) reported that increasing sp1 levels promotes the expression of wHtt, resulting in alleviating cellular toxicity induced by aggregated mHtt.

However, Qiu et al. (2006) demonstrated increased levels of sp1 in neuronal-like PC12 cells expressing mHtt. The pro-apoptotic role of sp1 might be associated with the REST promoter. Activation of REST by mHtt is mediated by sp1 in NG108 neuronal-like cells (Ravache et al., 2010). Mithramycin, a gene-selective sp1 inhibitor, extends the lifespan of *Drosophila* and mouse models of HD (Ferrante et al., 2004; Sleiman et al., 2011).

In brief, the relationship between sp1 and the pathogenesis of HD remains controversial. Sleiman found that the role of mithramycin in the development of HD is related to inhibiting oncogene expression. As reviewed by Kumar et al. (2014), the role of sp1 might be highly gene-dependent.

#### TATA-Box-Binding Protein

TBP, a part of RNA polymerase II, TFIID, increases in the postmortem HD brain (van Roon-Mom et al., 2002) and *in vitro* (Sinha et al., 2010). The TFIID complex decreases, whereas soluble TBP increases in the BACHD mice model (Yu-Taeger et al., 2017). No pathological TBP expansion is found in HD patients (Mariani et al., 2016). The toxicity of mHtt is enhanced in a fly model with a dysfunctional TBP (Hsu et al., 2014).

#### p53

Bae et al. (2005) confirmed that tumor suppressor p53 provides additional pro-apoptosis and might be dysregulated in HD; thus, promoting cell death and neurodegeneration. mHtt interacts with p53 and the p53 level increases in the brains of mHtt transgenic mice and patients with HD. They also found that genetic deletion of p53 suppresses neurodegeneration in mHtt-transgenic flies and neurobehavioral abnormalities of mHtt-transgenic mice. p53 upregulates HD promoter activity, as well as the level of wHtt mRNA and protein (Feng et al., 2006; Ryan et al., 2006).

P53 and the target molecules are upregulated in both cerebral cortex and striatum of HD patients (Kim et al., 2016). One study observed a significant increase in phosphorylation of p53 at serine-15 and total protein p53 expression in human HD induced pluripotent stem cells (iPSCs) (Tidball et al., 2016). However, p53 protein and mRNA are dysregulated in HD iPSCs (Szlachcic et al., 2015). Furthermore, mHtt knockdown rescues p53 deregulation in mouse iPSCs and NSCs (Szlachcic et al., 2017).

A further study confirmed an interaction between the post-translational modification of p53 and mHtt. mHtt may cause an increase of p53 phosphorylation on ser46, thereby inducing the expression of apoptotic target genes (Mantovani et al., 2007). Inhibiting ser46 phosphorylation prevents mHtt-dependent apoptosis of neuronal cells; thus, providing a potential therapeutic strategy for HD (Grison et al., 2011).

To conclude, p53 protein level and p53 phosphorylation increase in several HD models. Inhibiting p53 phosphorylation might be helpful in the treatment of HD.

#### Nuclear Factor κ Light-Chain-Enhancer of Activated B Cells

Nuclear factor κ light-chain-enhancer of activated B cells (NF-κB) is a family of DNA-binding proteins that are important transcription factors involved in immune and inflammatory responses, as well as in cell survival and apoptosis. Loss of Htt function and the HD mutation slows the rate of movement of NF-κB from the synapse to the nucleus (Marcora and Kennedy, 2010). The mammalian NF-κB family is comprised of five members: p65/RelA, RelB, p50/NF-κB1, p52/NF-κB2, and c-Rel. NF-κB is mainly composed of a heterodimer of p65 and p50 subunits (Zhang et al., 2013). RelA is downregulated by mHtt in PC6.3 neuronal cells (Reijonen et al., 2010). A similar result was observed in the R6/2 HD mouse striatum and in the caudate and putamen of late-stage HD patients (Laprairie et al., 2014). P50 exhibits a neuroprotective role in striatal neurons following 3-NP exposure (Yu et al., 2000).

The NF-κB pathway is also associated with neurodegeneration in non-neuronal HD cells. Enhanced activation of RelA is observed in astrocytes of a HD mouse model and in patients with HD (Hsiao et al., 2013). mHtt increases NF-κB activity in HD patient myeloid cells (Träger et al., 2014).

A non-coding SNP, rs13102260: G > A, in a NF-κB binding site modulates the binding of NF-κB and reduces transcriptional activity of the Htt gene promoter (Bečanović et al., 2015).

The NF-κB pathway inhibitor EVP4593 protects medium spinal neurons in the striatum of YAC128 mice against glutamine toxicity (Wu et al., 2011). Another antagonist, natrium diethyl dithio carbamate trihydrate restores 3-NP induced-symptoms of HD in rats (Gupta and Sharma, 2014). Another study found a protective role for ethyl pyruvate (EP), a pyruvate derivative, as it increased the survival rate in animal models of several diseases and is related to the inhibition of the NF-κB pathway in the striatum in a 3-NP-induced mouse model of HD (Jang et al., 2014). Sulforaphane might effectively attenuate 3-NP-induced striatal toxicity by inhibiting the NF-κB pathway (Jang and Cho, 2016).

In conclusion, RelA is down-regulated in HD neuronal cells, while enhanced activation of RelA level is observed in HD non-neuronal cells. Inhibiting the NF-κB pathway might also be helpful in the treatment of HD.

### Post-translational Modifications (PTMs)

Many groups have confirmed the role of PTM in the pathogenesis of HD, such as acetylation, phosphorylation, methylation, ubiquitination, and small ubiquitin modifier protein (SUMO) modification (Francelle et al., 2017; Sambataro and Pennuto, 2017), mainly at the 17 amino acid of the N-terminal Htt.

#### Acetylation and Histone Deacetylase Inhibitors

Acetylation has been best characterized, and a large number of groups have focused on the therapeutic role of histone deacetylase (HDAC) inhibitors. Several groups assumed early on that altered histone acetylation might contribute to HD when scientists discovered that histone acetyltransferases (HAT), such as CBP, were recruited into mHtt aggregates, and an HDAC inhibitor increased histone acetylation; thus, improving the phenotype in several animal models of HD (Steffan et al., 2001; Chuang et al., 2009).

Lysine residues (K6, K9, and K15) in the 17 amino acids of the N-terminal Htt are significantly acetylated, which retards fibril formation, promotes globular aggregation, and induces binding to lipids (Chaibva et al., 2016). Acetylated K6, but not K9 or K15, reverses the inhibition of threonine3 phosphorylation; thus, inhibiting aggregation of mHtt (Chiki et al., 2017). Acetylation at K444 promotes clearance of mHtt through autophagy (Jeong et al., 2009). Then, the expression of acetylated histones decreases significantly in cells from the postmortem HD brain (Yeh et al., 2013).

Different types of HDAC inhibitors can be used to increase acetylation, which is beneficial in the treatment of several human diseases (Schölz et al., 2015). Several preclinical studies have focused on the protective role of HDAC inhibitors, including sodium butyrate, phenylbutyrate, suberoylanilide hydroxamic acid (SAHA), and LBH589 *in vivo* and *in vitro* models. These models simulate the human HD pathology more closely. However, few clinical outcomes have been found, possibly because of the slow progression of HD.

RGFP966, a benzamide-type HDAC inhibitor, shows direct beneficial effects on HD disease phenotypes in N171-82Q transgenic mice (Jia et al., 2015). In the same HD mouse model, RGFP966 is associated with activation of glial cells and astrocytes (Jia et al., 2016). HDAC inhibitor 4b prevents the formation of mHtt aggregates in the brains of N171-82Q mice (Jia et al., 2012). SAHA may slow the progression of HD pathogenesis (Steffan et al., 2001) by inhibiting HDAC (Mielcarek et al., 2011). Phenylbutyrate exerts significant effects on survival and ameliorates degeneration in the N171-82Q HD mouse model (Gardian et al., 2005) and Sodium butyrate has similar effects in R6/2 mice (Ferrante et al., 2003). Trichostatin A, sodium butyrate, and sodium phenylbutyrate rescue cell viability in an HD striatal cell model (Naia et al., 2017). A phase II clinical trial with sodium butyrate found that 12–15 g/day is safe and well-tolerated in patients with HD (Hogarth et al., 2007). Lithiumcan induces HDAC1 degradation; thus, decreasing cytotoxicity (Wu et al., 2013). Chopra reported that the non-selective HDAC inhibitor LBH589 plays a neuroprotective role in both fragmented and full-length models of HD (Chopra et al., 2016).

Overexpression of sirtuin1 (Sirt1), a non-classical type of HDAC inhibitor, improves motor function, reduces brain atrophy, and attenuates mHtt-mediated metabolic abnormalities in both fragmented and full-length HD mouse models (Jiang et al., 2011). The Sirt1 activator resveratrol (RESV) decreases histone H3 acetylation at lysine 9 and improves motor coordination in the YAC128 mice model (Naia et al., 2016). RESV protects against peripheral deficits in the N171-82Q HD mice model (Ho et al., 2010). The small molecule sirtuin1 activator SRT2104 improves motor function and extends life span in the N171-82Q HD mouse model (Jiang et al., 2014).

The Sirt1 inhibitor nicotinamide also increases BDNF mRNA levels and improves motor deficits (Hathorn et al., 2011). Another selective Sirt1 inhibitor, selisistat, plays a neuroprotective role in *Drosophila* and R6/2 mice models, as well as in HEK293 cells (Smith et al., 2014). Selisistat is safe and well-tolerated when administered to patients with early stage HD. Süssmuth et al. (2015) failed to find any beneficial or adverse effects on any of the clinical outcome measures and they thought these results were related to the slow progression of HD. The Sirt2 inhibitor AK-7 ameliorates HD pathogenesis in R6/2 mice (Chopra et al., 2012). The bioactive sirt2 inhibitor MIND4 displays a similar role in *ex vivo* brain slices and in an *in vivo Drosophila* model of HD (Quinti et al., 2016). However, another group also found that inhibiting sirt2 had no effect on HD progression in the R6/2 mice model (Bobrowska et al., 2012).

#### Phosphorylation

Phosphorylation of Htt decreases during the pathogenesis of HD and mainly affects serine and threonine residues, and different results may occur in different residues. Phosphorylation on serine 421 of Htt may promote its clearance by the ubiquitin–proteasome system (UPS) and prevent striatal neurodegeneration caused by mHtt (Kratter et al., 2016). The level of phosphorylated mHtt decreases at serine 13 and 16 of the 17 amino acid N-terminal Htt (Gu et al., 2009; DiGiovanni et al., 2016). Phosphorylation at threonine 3 is reduced in HD postmortem tissue and the mouse models with a possible impact on aggregation, since this phosphorylation can affect confirmation and decrease mHtt aggregation (Cariulo et al., 2017).

#### Methylation

Arginine methylation, an important part of PTM, is very important in the pathogenesis of HD. The compensation of protein arginine methyltransferase 5, an enzyme mediating arginine demethylation, reverses the toxic effect of mHtt in HD pathogenesis (Ratovitski et al., 2015).

#### Ubiquitination

Several studies have confirmed the role of ubiquitination in the pathogenesis of HD. Ubiquitination of misfolded proteins is mediated by different lysine (K) residues in ubiquitin and alters the levels of toxic proteins. Ubiquitination involves the concerted actions of three enzymes: ubiquitin-activating (E1), ubiquitin-conjugating (E2), and a number of ubiquitin-ligating (E3) enzymes. mHtt aggregation has been reported in the cortex and striatum of the postmortem HD brain, consistent with the expression of ubiquitin (DiFiglia et al., 1997). Ubiquitinated mHtt aggregates are found in several brain areas in R6/2 HD mice (Gong et al., 2012). The UBB^+1^ protein, a frameshift mutation form of ubiquitin, accumulates in the brain of postmortem HD patients. It was found that UBB^+1^ induces aggregate formation of expanded polyQ in SH-SY-5Y neuroblastoma cells (de Pril et al., 2004). Although UBB^+1^ transgenic mice did not have a shorter life span in a later study, they were more vulnerable to toxic effects of expanded polyQ proteins (de Pril et al., 2010). The mHtt protein is ubiquitinated via K48 or K63 of ubiquitin, and triggered degradation is related to the UPS. K48-mediated ubiquitination promotes degradation, whereas K63-mediated ubiquitination accelerates aggregation (Bhat et al., 2014). Absence of the K48-specific E3 ligase Ube3a accelerate the disease phenotype and explain the shorter lifespan in HD mice (Maheshwari et al., 2014). WWP1, an E3 ligase, can ubiquitinate mHtt at K63 and thus reduce mHtt degradation through the ubiquitin–proteasome pathway (Lin et al., 2016). E2-25K modulates aggregation and toxicity of mHtt and E2-25K mutants can reduce polyglutamine-induced cell death (de Pril et al., 2007). A deficiency in Ube2W, an E2 ligase, that ubiquitinates substrates at their N-terminal, decreases mHtt aggregate formation and reduces mHtt-induced cytotoxicity (Wang B. et al., 2018). The levels of monoubiquitylated histone H2A (uH2A) reportedly increase in the R6/2 HD mouse striatum by several groups (Bett et al., 2009). One study suggested that the increase in uH2A might affect the phenomenon rather than transcriptional dysregulation in HD (McFarland et al., 2013).

#### Small Ubiquitin Modifier Protein Modification

Some studies have focused on the relationship between SUMO modification and the development of HD. SUMO modification at K6 and K9 in mHtt is reduced in mutant HD cell models (O’Rourke et al., 2013). Overexpression of the SUMO E3 ligase PIAS1 exacerbates mHtt-associated phenotypes and aberrant protein accumulation (Ochaba et al., 2016). Rhes, a small guanine nucleotide-binding protein (G-protein) selectively localizes to the striatum, binds to mHtt, and elicits its SUMOylation, which is associated with mHtt disaggregation and cell death (Subramaniam et al., 2009).

## Spinocerebellar Ataxia

### SCA1

SCA1 is an autosomal dominant progressive neurodegenerative disease characterized by ataxia, ophthalmoparesis, and a variable degree of motor weakness. Symptoms usually begin during the third or fourth decade of life and the disease gradually worsens, often resulting in complete disability and death 10–20 years after onset. Loss of Purkinje neurons within the cerebellum is found in patients with SCA1, which might be important in the development of the disease (Ingram et al., 2016).

SCA1 is caused by a CAG expansion encoding a polyQ expansion in the protein ataxin-1. Sp1 is an important transcription factor in HD that interacts with ataxin-1 and the interaction decreases when Atxn1 contains an expanded polyQ tract (Goold et al., 2007).

PolyQ expansion of mutant ataxin-1 increases the interaction with poly-Q tract-binding protein-1 (PQBP-1) which, in turn, stimulates PQBP-1 binding to RNA polymerase II (Pol II) and reduces Pol II phosphorylation and transcription (Okazawa et al., 2002). Transgenic mice of human PQBP-1 show a late-onset and gradually progressive motor neuron disease-like phenotype, which may be related to neurogenic muscular atrophy observed in patients with SCA1 (Okuda, 2003).

Ataxin-1 binds HDAC leading to strong transcriptional repression. The complete loss of HDAC results in progressive neurodegeneration in Purkinje cells, the most relevant cell type in SCA1 (Venkatraman et al., 2014). Ubiquitylation at K589 of ataxin-1 by the ubiquitin-conjugating enzyme UbcH6 decreases in mutant ataxin-1 cells and increases aggregation (Kang et al., 2015).

### SCA2

SCA2 is characterized by slowly progressive cerebellar ataxia and dysarthria with ocular findings (such as nystagmus and slow saccades). Excessive CAG repeats in the exon 1 of the ATXN2 gene in patients with SCA2 cause expansion of a polyQ domain in the ataxin-2 protein (Halbach et al., 2017). Ataxin-2-mutant mice exhibit an early pathological motor phenotype, resulting in a loss of molecular layer volume and aggregate formation in cerebellar Purkinje cells (Hansen et al., 2013).

Transcription of ataxin-2 can be activated by the interaction between ataxin-2 and the krüppel-associated box-containing zinc-finger transcriptional regulator, which participates in transcriptional repression of RNA polymerase as well as binding and splicing of RNA (Hallen et al., 2011).

### SCA3

SCA3, also known as Machado–Joseph disease, is the most common inherited ataxia that is also caused by CAG expansion in the deubiquitinase ataxin-3, coded by the ATXN3 gene (reviewed in Costa Mdo and Paulson, 2012). Wild-type ataxin-3 reduces the accumulation of pathogenic protein and suppresses toxicity in *Drosophila* with SCA3 (Tsou et al., 2015).

Transcriptional disruption and post-translational modifications, which play an important role in the pathogenesis of HD, are also involved in the development of SCA3. Inactivating ataxin-3 increases protein ubiquitination in a mouse model (Schmitt et al., 2007). *Drosophila* Myc (a homolog of the human cMyc proto-oncogene) suppresses polyQ toxicity by alleviating the cellular level of CBP and improving histone acetylation in a *Drosophila* model of SCA3 (Singh et al., 2014). Downregulation of TBP activity enhances retinal degeneration in SCA3 fly models (Hsu et al., 2014).

P53 might be acting as a substrate for ataxin-3, as polyQ-expanded ataxin-3 enhances interaction and deubiquitination catalytic activity of p53 and caused p53-dependent neurodegeneration in zebrafish brains and in the substantia nigra, pars compacta, or striatum of a transgenic SCA3 mice model (Liu et al., 2016). Activation of p53 by mutant ataxin-3 is also related to phosphorylation of p53 at ser15 residue in the SCA3 transgenic mice model. The p53 inhibitor pifithrin-α ameliorates neuronal death in the pontine nuclei of SCA3 transgenic mice (Chou et al., 2011b). Caffeic acid and RESV decrease neuronal cell death by reducing p53 and NF-κB activation in *Drosophila* and SCA3 cell models (Wu et al., 2017). HDAC inhibitors have been proven to be effective in SCA3 models. Ataxin-3 represses transcription by recruiting HDAC in SCA3 cell lines and human brain tissue (Evert et al., 2006). Histones H3 and H4 are hypo-acetylated in the cerebellum of SCA3 transgenic mice. The HDAC inhibitor sodium butyrate ameliorates ataxic symptoms and improves survival rate of SCA3 transgenic mice (Chou et al., 2011a; Lin et al., 2014). Divalproex sodium, an HDAC inhibitor, rescues the hypoacetylation levels of histone H3 and attenuates cellular cytotoxicity in the SCA3 cell model (Wang Z.J. et al., 2018). Another HDAC inhibitor, valproic acid (VPA), produces similar results in *Drosophila* and SCA3 cell models (Yi et al., 2013). Lin et al. (2014) reported that VPA enhances CREB-dependent transcriptional activation in PC12 cell models of MJD. Lei et al. (2016) displayed the clinical safety and efficacy of VPA treatment in patients with SCA3. They reported that patients with SCA3 can tolerate an 800 mg single oral dose of VPA, a multi-dose of 800 mg/day (400 mg, bid), or 1,200 mg/day (600 mg, bid) for 12 weeks. The latter two multi-dose treatments significantly improved locomotor function, which reveals that VPA might be a potential drug to treat SCA3.

### SCA6

PolyQ expansion in the α1A subunit (α1ACT), a CACNA1A gene, influences P/Q-type channel function and is associated with the dominantly inherited conditions of migraine, epilepsy, and episodic and progressive ataxia called SCA6 (Rajakulendran et al., 2012). Few studies have been conducted on the transcriptional dysfunction in SCA6. The expression of several target genes, such as TBP-associated factor 1, is impaired in α1ACT^-/-^ mice (Du et al., 2013).

### SCA7

SCA7 is a neurodegenerative disease primarily affecting the brainstem, retina, and Purkinje cells of the cerebellum (Ström et al., 2005). The disease is caused by polyQ expansion in ataxin-7, an integral component of the mammalian Spt/Ada/Gcn5 acetylase (SAGA)-like complexes, the TATA-binding protein-free TAF-containing complex, and the SPT3/TAF9/GCN5 acetyltransferase complex (STAGA) (Helmlinger et al., 2004).

CBP and p53 play roles in the pathogenesis of SCA7. Similar to ataxin-3, mutant ataxin-7 regulates activation of p53. Wang et al. (2010) reported that mutant ataxin-7 enhances phosphorylation of p53 at the ser15 residue, resulting in p53 activation and promoting neuronal apoptosis in a transgenic mice model. However, Ajayi et al. (2015) found that p53 and mutant ataxin-7 co-aggregate and reduce transcriptional activity of p53, leading to a decrease in the key p53 target proteins in a PC12 cell model of SCA7. Ström et al. (2005) demonstrated that the transcription mediated by CBP was repressed by expanded ataxin-7.

Histone deacetylase inhibitors and acetylation also participate in the regulation of SCA7. GCN5 is the HAT catalytic subunit of SAGA. Reducing GCN5 expression may accelerate both cerebellar and retinal degeneration in a mouse model of SCA7 (Chen et al., 2011). This phenomenon might be caused by direct inhibition of GCN5 HAT activity by polyQ-expanded ataxin-7 *in vivo* and *in vitro* (Burke et al., 2013). PolyQ expansion may decrease gene transcription associated with ataxin-7 and is correlated with increased levels of histone H2B monoubiquitination. Treatment with the HDAC inhibitor trichostatin A can rescue the effects of mutant ataxin-7 protein (McCullough et al., 2012).

miR-124 is a miRNA regulated to the expression of REST and BDNF, as we reviewed before. Tan et al. (2014) reported that ataxin-7 is required to initiate transcription of miR-124. MiR-124 also mediates post-transcriptional crosstalk between the ataxin-7 gene and lnc-SCA7, a conserved long coding RNA.

### SCA17

SCA17 is an autosomal dominant cerebellar ataxia caused by expansion of polyQ within TBP. Loss-of-function mutations of TBP in *Drosophila* reveal SCA17-like neurodegeneration (Hsu et al., 2014). Flies expressing a human expanded polyQ TBP protein exhibit progressive neurodegeneration, late-onset locomotor impairment, and a shortened lifespan (Ren et al., 2011). Mutant TBP in muscle cells also causes muscle degeneration, which is a characteristic of patients with SCA17 (Huang et al., 2015). Blocking NF-κB restores the neurotoxicity caused by mutant-TBP in astrocytes (Yang et al., 2017).

## Spinal and Bulbar Muscular Atrophy

Spinal and bulbar muscular atrophy, or Kennedy’s disease, is an X-linked, adult-onset disease with slow progressive weakness of the bulbar and extremity muscles due to degenerative motor neurons in the brainstem and spinal cord. SBMA is a male-specific disease, and is caused by the polyQ expansion in the androgen receptor (AR) (Rocchi et al., 2016).

The HDAC inhibitor sodium butyrate improves motor impairment and ameliorates the neurological phenotype in the SBMA mouse model (Minamiyama, 2004). Sirt1, a class III HDAC, protects motor neurons expressing the polyQ-expanded AR. Sirt1 has been demonstrated to reduce AR acetylation at lysine residues 630, 632, and 633 (Montie et al., 2011).

Arginine methylation also takes part in the development of SBMA. Protein arginine methyltransferase 6 (PRMT6) acts as a co-activator of the AR and the interactions between PRMT6 and AR are significantly enhanced in cell and fly models of SBMA. The inhibitor of PRMT6 can reduce toxicity of the mutant AR (Scaramuzzino et al., 2015).

SUMOylation plays a role in the pathogenesis of SBMA. In both cell and mouse models of SBMA, decreasing SUMOylation restores mutant AR function and dissociates the pathogenic role of AR dysfunction (Chua et al., 2015).

## Dentatorubral-Pallidoluysian Atrophy

Dentatorubral-pallidoluysian atrophy (DRPLA) is an autosomal dominant progressive neurodegenerative disease that results in intellectual deterioration and various motor deficits, including ataxia, choreothetosis and myoclonus. DRPLA is caused by an abnormal expansion of CAG repeats within the atrophin-1 protein (Suzuki et al., 2012). Sodium butyrate ameliorates the histone acetylation defects, improves motor performance, and extends the average life span in transgenic mice with mutant atrophin-1 (Ying et al., 2006).

## Conclusion and Perspective

Transcriptional disruption and post-translational modifications have been well-established as important pathological processes in polyglutamine diseases. Transcriptional disruption may interact with post-translational modifications. For example, the HAT CBP also acts as a CREB-binding protein and can take part in transcriptional dysfunction in HD, whereas post-translational modification affects transcriptional regulation, mutant ataxin-3 and mutant ataxin-7 could cause phosphorylation and ubiquitination of p53, resulting in p53 transcriptional regulation (Wang et al., 2010; Chou et al., 2011b; Ajayi et al., 2015; Liu et al., 2016). In addition, the transcription factors also interact with each other. Sp1 mediates activation of REST by mHtt (Ravache et al., 2010).

As dysfunction of transcription and post-translational modifications has been found in polyQ diseases during the last decades, huge efforts have been made by researchers. Beneficial effects have been observed in various preclinical polyQ disease models, through corrections in transcriptional dysregulation and post-translational modifications. PDE inhibitors and HDAC inhibitors are the most widely studied agents. A large number of studies have confirmed their therapeutic value in polyQ diseases, while the precise mechanism of action has yet to be determined. Safety and efficacy of HDAC inhibitors and PDE inhibitors are currently being tested. However, the results are not as expected. Vast problems need to be solved. The specificity of compounds altering transcription remains an important concern. Numerous cell-based and whole-animal studies have shown that the compounds have diverse biological activities that impact physiological and developmental functions. This problem has recently been reviewed for HDAC inhibitors (Millard et al., 2017). The differences between the observed effects in cellular or animal models compared to the effects in humans are huge problems. Many reasons could contribute to the observed differences. For example, drug delivery to the brain remains one of the major obstacles. Unique brain metabolism also needs to be considered, as the pharmacokinetic and pharmacodynamics characteristics of drug candidates are often different in brain tissues compared to those in other organs. Evidently, we might need to find better models, which are more suitable to mimic the action in the human brain.

## Author Contributions

CX contributed to drafting and revising the manuscript. SZ contributed to drafting and modifying the table. XD and SM contributed to drafting and modifying the figure. SC contributed to drafting and revising the manuscript. All of the authors approved the final version of the manuscript and agreed to be accountable for all aspects of the work in ensuring that questions related to the accuracy or integrity of any part of the work are appropriately investigated and resolved.

## Conflict of Interest Statement

The authors declare that the research was conducted in the absence of any commercial or financial relationships that could be construed as a potential conflict of interest.
